# Effect of Behavioral Activation for Women with Postnatal Depression: A Systematic Review and Meta-Analysis

**DOI:** 10.3390/nursrep14010007

**Published:** 2024-01-03

**Authors:** Engida Yisma, Sandra Walsh, Mary Steen, Richard Gray, Shaun Dennis, Marianne Gillam, Nayana Parange, Martin Jones

**Affiliations:** 1 Department of Rural Health, Allied Health & Human Performance, University of South Australia, Adelaide, SA 5000, Australia; sandra.walsh@unisa.edu.au (S.W.); mary.steen@curtin.edu.au (M.S.); r.gray@latrobe.edu.au (R.G.); shaun.dennis@unisa.edu.au (S.D.); marianne.gillam@unisa.edu.au (M.G.); martin.jones@unisa.edu.au (M.J.); 2 IIMPACT in Health, University of South Australia, Adelaide, SA 5000, Australia; nayana.parange@unisa.edu.au; 3 Department Nursing, Faculty of Health Sciences, Curtin University, Perth, WA 6102, Australia; 4 School of Nursing and Midwifery, La Trobe University, Melbourne, VIC 3086, Australia; 5 Flinders and Upper North Local Health Network, Whyalla, SA 5600, Australia; 6 Allied Health & Human Performance, University of South Australia, Adelaide, SA 5000, Australia

**Keywords:** behavioral activation, postnatal depression, women, systematic review, meta-analysis

## Abstract

Evidence shows that behavioral activation (BA), a simple form of psychological therapy, is as effective as the more complex psychological therapy—cognitive behavioral therapy (CBT)—in treating general depression. However, it remains unclear whether BA when compared with treatment-as-usual (TAU) has greater contributions in reducing postnatal depression. This systematic review compared the effect of BA versus TAU in reducing depression symptoms among postnatal women. Five databases (MEDLINE, Embase, Emcare, Cochrane Library, and PsycINFO) were searched. Risk of bias was assessed using the Cochrane Collaboration’s ‘risk-of-bias 2 tool’. A random-effects meta-analysis was conducted to examine the effect of BA on postnatal depression. Of 2844 initial studies, only two randomized control trials (RCTs) met the inclusion criteria. The overall quality of evidence of these two RCTs was low. When compared to TAU, meta-analysis showed that BA was associated with reduced depression symptoms in postnatal women (standard mean difference −0.56; 95% confidence interval −0.76 to −0.37). This review suggests that BA might be more effective than TAU for alleviating postnatal depression. However, due to concerns about evidence quality, these findings should be interpreted cautiously.

## 1. Introduction

Postnatal depression (PND) is a non-psychotic disorder affecting about 17.2% of the world’s population following childbirth [1]. Postnatal depression has impacts on the relationship between mother and infant, partner, and family [2]. It is widely recognized that women experiencing postnatal depression will struggle in their role of motherhood and this can have negative consequences on long-term emotional and cognitive development of the child [3]. To compound this, recognition of PND amongst health care professionals is poor [4]. Woman with PND can face additional barriers to accessing services, such as getting to services due to the baby’s routine, which does not align with office hours, and issues of stigma from health services [5]. There is also a reluctance in some women with PND to seek help or treatment [6,7].

Although pharmacological interventions, such as antidepressants, are effective in treating women with PND, many women prefer psychotherapy due to fear of side-effects/addiction, particularly during breastfeeding [8]. The National Institute for Health and Clinical Excellence (NICE) clinical guidelines recommends a high-intensity psychological intervention, such as cognitive behavioral therapy (CBT), for a woman with moderate or severe depression in the postnatal period [9]. Evidence from a 2018 systematic review and meta-analysis of 20 randomized control trials involving 3623 participants showed that CBT is effective in improving the symptoms and arresting the progression of PND [10]. The review found that CBT was associated with a significant decrease in the Edinburgh Postnatal Depression Scale (EPDS) among postnatal depressed women in the short term (mean difference −2.86, 95% CI −4.41 to −1.31) and long term (mean difference −1.68, 95% CI −1.81 to −1.56). However, CBT is complex and costly [11]. As the ‘cognitive’ component of CBT focuses on teaching skills for challenging negative thoughts, CBT involves a lengthy period of training of the therapist [12] and requires specialist qualifications as mental health workers (MHWs) to deliver the therapy. On the other hand, BA, a component of CBT, has been used for decades as the “behavioral” component of CBT or as stand-alone treatment for depression [13]. The aim of BA is to reverse the cycle of depression by monitoring mood and increasing engagement in valued activities [14,15].

A 2016 randomized control trial by Richards et al. [16] examined the clinical efficacy and cost-effectiveness of BA compared with CBT for adults with depression. They found that BA is as effective as CBT and can be delivered by junior MHWs with less intensive and costly training. This means that modifying behavior may be enough to improve depression and it may be unnecessary to directly challenge negative thinking using CBT. BA, thus, can be used as a preferred psychological treatment option, as it is suitable for delivery without the need for costly and highly trained professionals. BA may be considered as an opportunity for women living in communities in which accessing specialist MHWs is challenging. Due to the simplicity of BA, other groups of health workers could be trained and prepared to practice BA.

## 2. Materials and Methods

### 2.1. Eligibility Criteria

Studies involving participants aged 18 years and older with postnatal depression were eligible. Postnatal depression must have been verified by any standardized measure of PND, such as the Edinburgh Postnatal Depression Scale (EPDS) [17,18,19]. We focused on studies involving BA as the primary treatment based on any type of delivery mode, including face-to-face or online individual/group sessions. BA was defined as a “behaviourally oriented time limited psychotherapeutic intervention” [12] (p. 2). The psychotherapeutic intervention needed to include the two core elements of BA, namely mood monitoring and activity scheduling. Peer-reviewed publications were reviewed and only studies published in English were included. Studies were not excluded based on sample size, follow-up period, or year of publication.

### 2.2. Search Methods

The following five electronic databases were searched: MEDLINE, Embase, Emcare, Cochrane Library, and PsycINFO. The population search terms related to postnatal depression included “postnatal depression”, “postpartum depression”, postpartum, postnatal, post-partum, and post-natal. The intervention search terms (keywords) related to behavioral activation included: “behavio* activation”, “behavio* therapy”, “activity schedul*”, “positive reinforce*”, “event schedul*”, “mood monitoring”, “behavio* treatment”, “behavio* intervention”, “behavio* modif*”, and “behavio* psychotherap*”. Three reviewers (EY, MY, and SW) completed the title and abstract, and full-text screening. A third reviewer (RG) was involved in resolving any conflicts. The comprehensive search strategy for each database is provided in the Supplementary Material (Tables S1–S5).

### 2.3. Data Collection and Extraction

A data extraction sheet was used to collect data from the included studies. The data were first extracted on 24 February 2022. The data extracted from each study included (1) basic information such as authors’ name, year of publication, the number of cases in each group of participants, average age, duration of follow-up time, outcome measure, and full intervention details (type, frequency, etc.), and (2) statistical data for the primary outcome (PND) such as odds ratio (OR)/relative risk (RR) and 95% confidence interval (CI) for dichotomous data, and standardized mean differences (with 95% CI) for continuous data. Two review authors (SW and MJ) extracted the data independently. When discrepancies arose between the reviewers, these were resolved by involving a third reviewer (RG). All the included studies reported the required data; therefore, it was not necessary to contact any of the authors.

### 2.4. Quality Assessment and Risk of Bias

The quality of each included trial was assessed using the Cochrane Collaboration’s ‘Risk of bias 2’ tool [20], which considers the following domains: (1) risk of bias arising from the randomization process, including allocation and randomization, (2) risk of bias due to deviations from the intended interventions, including blinding of participants and people delivering the interventions, (3) risk of bias due to missing outcome data, (4) risk of bias in measurement of the outcome, including blinding of outcome assessors, and (5) risk of bias in the selection of reported results.

Two review authors (EY and MJ) independently assessed the risk of bias in included trials and discussed any disagreements with a third review author (RG). All ‘Risk of bias’ data were presented graphically using robvis (visualization tool) [21], and narratively in the text.

### 2.5. Data Synthesis and Statistical Analysis

We provided a narrative synthesis of the findings from the included studies. A meta-analysis was conducted to provide a consolidated estimate of the effect of BA versus TAU on reducing postnatal depression symptoms in women who have given birth in the past 12 months. We synthesized the information regarding reduction in the level of postnatal depression symptoms following behavioral activation therapy versus TAU by undertaking a random-effects meta-analysis using inverse variance weighting. We presented pooled effect size (standardized mean difference (SMD)) with 95% confidence interval (CI) and used forest plot with I^2^ to display statistical heterogeneity. All analyses were conducted using STATA/SE version 17.0 (Stata Corporation, College Station, TX, USA).

The review protocol was registered with Open Science Framework (Registration DOI: 10.17605/OSF.IO/PNQ3D). Reporting was consistent with the 2020 Preferred Reporting Items for Systematic Reviews and Meta-Analyses (PRISMA) [22]. We provided the PRISMA checklist in the Supplementary Material Table S6.

### 2.6. Patient and Public Involvement

There was no patient or public involvement in this study. Future research that considers using BA with women experiencing PND will engage with patients and the public.

## 3. Results

**Figure 1:**
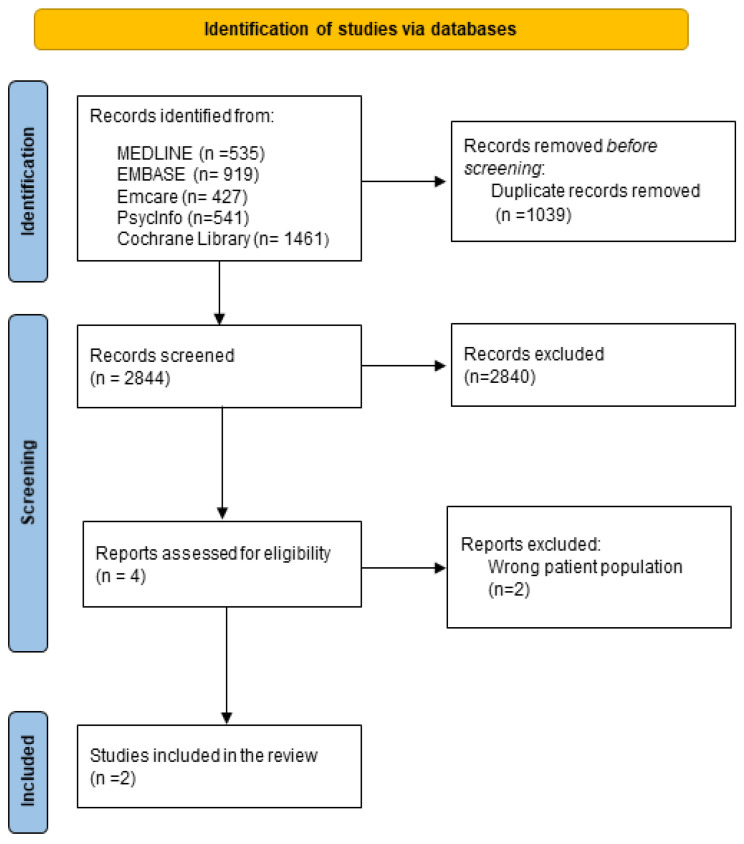
PRISMA 2020 flow diagram for the entire review.

The flow of publications during the review process is shown in Figure 1. The initial search identified 3883 citations, of which 1039 were duplicates and removed. A total of 2840 citations were excluded during the title and abstract screening. The full texts of two studies were reviewed but rejected. Both studies included the wrong patient population. Two studies were eligible to be included in the final review.

### 3.1. Descriptive Characteristics of Studies, Population, Intervention, and Outcomes

The first study conducted by O’Mahen et al. in 2013 [23] recruited women with PND via an online website. Women who scored 12 and above on the EPDS (indicating moderate to severe depression) were invited to complete an 11 session weekly online training program in BA. Of the 462 women who were invited to participate, 181 were entered for analysis. Ninety-five women completed 11 sessions. The average length of the sessions was not reported, nor was the expected minimal dose of BA. Moreover, the authors did not clarify how engagement with the module was determined, whether it was opening the module or demonstrating some other engagement. The women allocated to BA reported meaningful improvements on the EPDS. The online treatment program followed the core principles of BA, based on the manual by Addis and Martell [24], and was modified for online delivery and to support women with PND. These modifications were informed by interviews with women with PND [25]. Modifications included exploring the challenges of finding alternative activities as a mother. The program occurred weekly via an online platform and was interactive in nature. Participants were provided the opportunity to complete the program over a 15-week period. It was reported that each online session lasted for 40 min. The program had links to Netmums cache of activities and a feature called “meet a mum”, which helped facilitate meetings with other mums in their area. Additionally, participants had access to a chat room facility via the online program.

The second study (O’Mahen et al. (2014)) [26] invited women with PND to complete 12 sessions of an online program in BA. O’Mahen et al. (2014) [26] followed a similar recruitment strategy as previously undertaken by O’Mahen et al. (2013), i.e., using banners on Netmums and chat groups. Participants were invited to participate if they scored 12 or more on the EPDS. O’Mahen et al. (2014) [26] extended the education program reported by O’Mahen et al. (2013) by increasing the number of sessions to 12 and delivering the sessions over five self-directed modules with an additional module on relapse prevention. The mean number of BA sessions participants completed was 6.74. O’Mahen et al. (2014) [26] retained the “meet a mum” feature and the chat room facility consistent with the first study by O’Mahen et al. (2013). In addition to this, the researchers introduced weekly support calls for the participants with a specialist MHW who completed five days of face-to-face training in BA. O’Mahen et al. (2014) [26] reported that each telephone conversation was 20–30 min in length, with 29 min being the average length of the weekly support calls. The focus of the conversation was on completing the treatment material from the previous session and overcoming any barriers to implementation. If weekly calls were missed, participants were actively followed up.

The control condition in both papers was reported as the same. The treatment-as-usual (TAU) condition was allowed to vary according to usual practice. Women in both groups had access to Netmums’ general depression chat room throughout the course of the study. This chat room was moderated by health visitors and parent supporters who provided email/chat room posting support and advice for depression [26].

O’Mahen et al. (2013) screened 1403 for eligibility, and 493 were excluded as they did not meet the eligiblity criteria or did not give consent. These researchers randomized 910 women, 448 to TAU and 462 to BA. At the 15-week follow-up, 162 women in the TAU group and 181 in the BA group were entered for analysis. O’Mahen et al. (2013) stated that at the 15-week assessment, fewer women in the BA group (*n* = 66/181) exceeded the depression cut off than in the TAU group (*n* = 91/162). In the second study, O’Mahen et al. (2014) screened 249 for eligibility, 166 did not meet the criteria, and 83 participants were randomized; 42 women were allocated to TAU and 41 to BA. At 17-week follow-up, there were 34 women in TAU and 38 in the BA group entered into the analysis.

Both papers used the EPDS to measure depression. O’Mahen et al. (2013) reported a decrease in mean depression scores from baseline to 15-week follow-up for both the BA group (19.46, standard deviation (SD) 3.81 baseline to 10.94, SD 5.57 follow-up) and the TAU group (19.44, SD 3.8 baseline to 14.28, SD 6.63 follow-up). The authors report ‘reliable and clinically significant improvement’ for 61.3% (*n* = 111/181) of participants in the BA group compared to the TAU group at 41.4% (*n* = 67/162), with a *p* < 0.001 for observed data only (odds ratio for improvement in comparing BA to TAU group was 2.17 (95% CI 1.40 to 3.37)).

O’Mahen et al. (2014) also reported a decrease in mean depression scores from baseline to 17-week post-randomization follow-up for both the BA group (20.24, SD 3.28 baseline to 11.05, SD 4.71 follow-up) and the TAU group (21.07, SD 4.0 baseline to 14.26, SD 5.1 follow-up). Additionally, O’Mahen et al. (2014) reported a large Cohen’s d effect size favoring those in the BA group for depression measured on the EPDS (−0.87, 95% CI −0.42 to −1.32). The researchers also noted a large effect size for work and social impairment, and anxiety scores for women in the BA group compared to those in the TAU group.

In both studies, it was noted that no harm or adverse events were reported. No information about care and support from other services provided to the research participants was identified. Both studies were not registered as a clinical trial but had ethics approval from the University of Exeter review committee.

### 3.2. Risk of Bias Assessment

**Figure 2:**
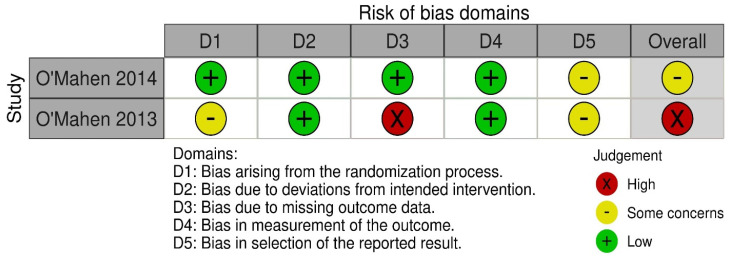
‘Risk of bias’ summary: review authors’ judgements about each risk of bias item for each included study [23,26].

**Figure 3:**
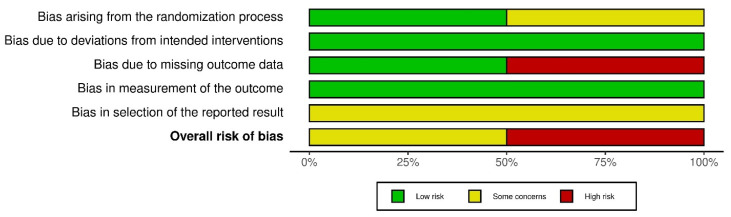
‘Risk of bias’ graph: review authors’ judgements about each risk of bias item presented as percentages across all included studies.

Of the two studies included in the review, one study was rated as having some concerns, while the other study was rated as having a high risk of bias in the overall assessment of risk of bias (see Figure 2 and Figure 3). Figure 2 shows the risk of bias assessment for five domains across the two included studies. For the domain of bias in the selection of the reported results, both studies were rated as having “some concerns”. Regarding bias arising from the randomization process, O’Mahen et al. 2013 was rated as having “some concerns” because the study did not provide sufficient details about the randomization methods used. For missing outcome data, O’Mahen et al. 2013 was rated as “high risk” because it had high attrition, while O’Mahen et al. 2014 was rated as “low risk”.

Figure 3 shows that across all bias domains, 50% were rated as “high risk” and 50% had “some concerns”. This reflects issues mostly around randomization methods, missing data handling, and selection of results for reporting.

### 3.3. Effects of BA versus TAU on Reducing Postnatal Depression

**Figure 4:**
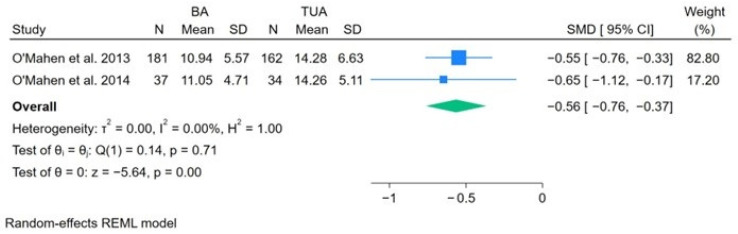
Meta-analysis showing the association between behavioral activation and postnatal depression [23,26]. The blue squares represent effect sizes from individual studies included in the meta-analysis. The green rhombus represents the pooled effect size across studies.

Figure 4 shows the random-effects meta-analysis results. Compared to treatment as usual, BA is associated with a decrease in postnatal depression symptoms in women by a mean of 0.56 SD (SMD −0.56; 95% CI, −0.76 to −0.37).

## 4. Discussion

This systematic review sought to identify and understand the published studies that examined BA and postnatal depression. Only two papers were located; therefore, caution needs to be exercised when interpreting the results. This is particularly so, as the studies were conducted by the same authors, using a similar online program, with similar study elements, such as participant recruitment. Nevertheless, we note the potential for BA to improve depression for women who may be experiencing postnatal depression.

### 4.1. Online BA with Support May Be Beneficial to Women with PND

BA is a third-wave psychological treatment that can be delivered via face-to-face or online platforms. Both studies included in this review [23,26] provided a rationale for conducting the studies via online delivery, stating that women were more likely to participate in an online program rather than face-to-face. As has been noted from previous studies [6,7], women in the postnatal period may be reluctant to engage in mental health support due to concerns about their parenting skills being criticized and the risk of having the baby taken from them. Both included studies [23,26] acknowledged this concern as a reason for pursuing delivery of BA online. Interestingly, there was a high attrition rate in the O’Mahen et al. (2013) study. Of the 468 allocated participants, only 95 completed the 11-session program. In the second study, women with PND were offered 12 sessions of online BA and supported by weekly phone calls from MHW [26]. Attrition rates were less detrimental in this study, with 41 participants enrolled onto the BA program, of which 37 entered for analysis. The introduction of weekly phone calls to support women with PND in the second study appeared to help the participants to better adhere to the program. It has been reported previously that generally, attrition rates for computerized CBT tend to be high [27,28], so perhaps the high attrition rate in the included studies is not too surprising. Therefore, when considering the possibility of utilizing online treatment programs for BA and PND as reported by O’Mahen et al. (2014) [26], additional support may be required from suitable prepared health care workers. The authors of this review are speculating that as BA is a much simpler treatment, adherence with computerized BA programs with ongoing support may increase completion of BA activities. This speculation may indicate that the inclusion of weekly contact from a BA specialist will be an important consideration for improving the retention of participants in online BA programs for women with PND.

### 4.2. Scalability of BA

Typically, specialist MHWs (psychologist/psychiatrist) deliver CBT and it requires substantial training; it is also complex and can be costly to deliver [12]. This can be a barrier to accessing appropriate psychological supports. This is particularly problematic for communities who experience social and economic disadvantages in which recruiting and retaining specialist MHWs is challenging. An advantage of BA is that it can be delivered by a broad spectrum of health workers, after a short period of education and training, which can be implemented as a continuing professional development. The efficacy of BA in treating depression has been demonstrated by previous systematic review [29]. To the best of the authors knowledge, there is no existing systematic review and meta-analysis that demonstrates the efficacy of BA using online delivery methods, and this would be worthy of future work. Equally, this review did not yield any clinical trials where BA was delivered face-to-face to women experiencing PND. This is an area for further investigation to demonstrate whether BA is effective in reducing depression symptoms in women with PND based on any type of delivery mode, including face-to-face or online individual/group sessions. The lack of evidence identified in this review suggests that further work is urgently required.

### 4.3. Effectiveness of BA

Our finding in the meta-analysis combining the two studies shows that behavioral activation may be more effective when compared to TAU in reducing depression symptoms in women with postnatal depression. Given our concerns about the quality of the evidence, this finding should be interpreted cautiously. However, it appeared that there was better completion when the online program was supported by weekly contact with a trained MHW. Therefore, this relational component when investigating the effectiveness of BA needs to be considered in future studies. No mention was made in either paper regarding the minimum dose of BA or the number of modules required to be completed in order to be considered as completing the BA program. This limitation makes it difficult to confirm or refute whether BA was effective for women with PND. Nevertheless, both studies included in this review reported improvements in depression symptoms on the EPDS for women in boths groups—BA and TAU. It was reported that there was more improvement for women who participated in BA [23,26], and Mahen et al. [26] noted a large effect size.

### 4.4. Clinical Governance

PND can be a serious mental health condition, often requiring specialist mental health service input [30]. It is noteworthy that over 500 women participated in the two clinical trials, living with a potentially serious mental health condition. In the two included studies, additional support provided by specialist mental health services was not identified. The evidence from the two studies indicates that it may be beneficial when using online BA sessions that these are supported with phone or face-to-face interactions with MHWs. The included studies did not report any distress protocols if participants became unwell and a cause for concern. Harm or any adverse outcomes were not reported during the trials and there was no mention of suicidality and follow-up of women who did not complete the sessions. Neither of these two studies were registered.

### 4.5. Strengths and Limitations of the Study

The strengths of this systematic review are that a review protocol was published on Open Science Framework (Registration DOI 10.17605/OSF.IO/PNQ3D). No changes were made to the protocol. This review used a rigorous search strategy and was guided by the PRISMA checklist [22].

In this review, the outcome of interest was reduction in depressive symptoms. The inclusion criteria included all studies that used experimental designs. The review focused on a single primary outcome, which may be viewed as a limitation. The researchers could have included studies examining the experiences of women using BA in treating PND or indeed other potential outcomes associated with BA as secondary outcomes. However, the researchers chose not to include other outcomes, as there is a considerable risk of selective reporting bias, where the study authors select, post hoc, the most interesting outcomes. Another notable limitation of our study is the inclusion of only two randomized control trials conducted by the same research team. This introduces a potential source of bias, as both trials share duplicated authorship and employ very similar intervention delivery methods and platforms. Moreover, there may be a risk of publication bias, as our review was limited to English language publications only. Additionally, no grey literature was examined, and therefore, there is the possibility of missed studies in this area.

## 5. Conclusions

While the effect of BA on depression is widely recognized, there is a lack of evidence and studies that have examined the effectiveness of BA for women with PND. There were only two peer-reviewed studies identified that met the review inclusion criteria. The meta-analysis of these two studies suggests that internet-based behavioral activation may be more effective when compared to treatment-as-usual in reducing depression symptoms in women diagnosed with postnatal depression. However, the findings should be interpreted cautiously, given our concerns about the quality of the evidence. Further investigation regarding the effectiveness of BA, whether it is provided by face-to-face or online interactions, for postnatal depression would be required.

## 6. Future Research

Due to the paucity of evidence, it is important to better understand what a model of BA for women experiencing PND, co-designed with women and key stakeholders, could look like. The review identified two papers which utilized an online program to support women with PND, one with additional telephone support. Therefore, the authors recommend that further research should be conducted to examine which models of delivery are most effective. For example, understanding the effectiveness and acceptability of standalone online BA self-help programs, online self-help programs with weekly support, and face-to-face delivery of BA is essential. In the absence of research in this area, it is important to understand how feasible it is for health care workers or lay workers to be educated and trained to deliver BA to women with PND or if women with PND can complete self-help online BA programs. Given that about 17.2% [1] of women who give birth develop PND, it would be interesting to investigate and explore if BA during pregnancy can help prevent PND after childbirth.

While this review acknowledges the potential of online programs to reach more people, it is important that research first demonstrates the efficacy of BA in reducing depression symptoms in women experiencing PND. There is an opportunity to deliver BA through a range of other modalities, such as individually or in groups, in person (face-to-face) or using telecommunication options, such as telephone, apps, online platforms, or videoconferencing. If the preference of postnatal women is to access and engage with online options, then consideration must be given to opportunities to improve participation, engagement, retention, completion, support, and referral as necessary.

The authors identified, when undertaking a search of study protocols, that a study has been registered to be conducted in Japan. A registered study protocol for a new trial in Japan was reported—Smart Mama [31]. The study protocol (issue date: 2019-Mar-01, original version 2019005NI-00) was registered at the UMIN Clinical Trial Registry (UMIN-CTR: ID UMIN 000036864). The authors of this review will be interested in this ongoing study’s findings to see if these confirm or refute the limited evidence identified and reported by the two included studies undertaken in the UK.

## Data Availability

The data that support the findings of this study are available on request from the corresponding author.

## Supplementary Materials

The following supporting information can be downloaded at: https://www.mdpi.com/article/10.3390/nursrep14010007/s1, Tables S1–S5: The comprehensive search strategy for each of the five databases. Table S6: PRISMA 2020 Checklist.

## Public Involvement Statement

No public involvement in any aspect of this research.

## Guidelines and Standards Statement

This manuscript was drafted against the Preferred Reporting Items for Systematic Reviews and Meta-Analyses (PRISMA) [22] for Systematic reviews research.
