# Supplementary material for: Effect of Behavioral Activation for Women with Postnatal Depression: A Systematic Review and Meta-Analysis
Source: Nurs Rep. 2024 Jan 3;14(1):78–88. doi: 10.3390/nursrep14010007 (PMC10801525; doi:10.3390/nursrep14010007)
Supplement: Supplementary file 1 [file nursrep-14-00007-s001.zip › Table S1-S6 Search strategy.pdf]

Search Strategy

Table S1: Ovid MEDLINE(R) ALL <1946 to October 12, 2021>

#	Query	Results from 13 Oct 2021
**1**	behavio* activat*.ti,ab,kf.	2,274
**2**	(behavio* adj3 (reinforce* or re-inforce*)).ti,ab,kf.	3,241
**3**	(behavio* adj2 (contracting or modif*)).ti,ab,kf.	10,401
**4**	reinforc*.ti,kf. or ((positive adj1 reinforc*) or (reinforc* adj3 (environment* or experience*))).ti,ab,kf.	26,509
**5**	(activit* adj2 schedul*).ti,ab,kf.	618
**6**	((pleas* or enjoyable or rewarding) adj3 (activit* or event?)).ti,ab,kf.	1,343
**7**	((operant or instrumental) adj (conditioning or learning)).ti,ab,kf.	3,544
**8**	(positive interaction* or avoida* coping or environmental contingenc* or contingency management).ti,ab,kf.	5,366
**9**	functional analysis.ti,ab,kf.	26,695
**10**	behavio*.mp. and (self adj (evaluat* or monitor*)).ti,ab,kf.	4,303
**11**	(behavio* adj (counsel* or intervention* or treatment* or therap* or psychotherap*)).ti,ab,kf.	43,027
**12**	(mood adj3 monitor*).ti,ab,kf.	307
**13**	Behavior Therapy/	29,091
**14**	or/1-13	140,903
**15**	depression/	132,979
**16**	depressive disorder/	74,245
**17**	mood disorders/	15,087
**18**	depressive disorder, major/	33,389
**19**	depressive disorder, treatment-resistant/	1,661
**20**	dysthymic disorder/	1,154
**21**	cyclothymic disorder/	750
**22**	(depress* or dysthymi* or cyclothymi* or low mood or mood disorder* or affective disorder*).ti,ab,kf.	523,367

**23**	or/15-22	567,714
**24**	Pregnancy/	919,510
**25**	Peripartum Period/	1,445
**26**	Postpartum Period/	27,673
**27**	Puerperal Disorders/	11,507
**28**	(pregnan* or ante natal or antenatal or post partum or postpartum or ante partum or antepartum or post natal or postnatal or peri partum or peripartum or peri natal or perinatal or intra partum or intrapartum or puerp* or after birth).ti,ab,kf.	781,080
**29**	or/24-28	1,202,530
**30**	23 and 29	28,370
**31**	Depression, Postpartum/	6,334
**32**	30 or 31	29,201
**33**	14 and 32	535

Table S2: Embase Classic + Embase <1947 to 2021 October 12>

#	**Query**	**Results from 13 Oct 2021**
**1**	behavio* activat*.ti,ab,kw.	2,847
**2**	(behavio* adj3 (reinforce* or re-inforce*)).ti,ab,kw.	4,055
**3**	(behavio* adj2 (contracting or modif*)).ti,ab,kw.	13,492
**4**	reinforc*.ti,kw. or ((positive adj1 reinforc*) or (reinforc* adj3 (environment* or experience*))).ti,ab,kw.	27,043
**5**	(activit* adj2 schedul*).ti,ab,kw.	912
**6**	((pleas* or enjoyable or rewarding) adj3 (activit* or event?)).ti,ab,kw.	1,861
**7**	((operant or instrumental) adj (conditioning or learning)).ti,ab,kw.	4,130
**8**	(positive interaction* or avoida* coping or environmental contingenc* or contingency management).ti,ab,kw.	6,604
**9**	functional analysis.ti,ab,kw.	32,790
**10**	behavio*.mp. and (self adj (evaluat* or monitor*)).ti,ab,kw.	5,579

**11**	(behavio* adj (counsel* or intervention* or treatment* or therap* or psychotherap*)).ti,ab,kw.	58,715
**12**	(mood adj3 monitor*).ti,ab,kw.	439
**13**	Behavior Therapy/	45,371
**14**	or/1-13	178,144
**15**	depression/	407,458
**16**	mood disorder/	46,710
**17**	major depression/	70,742
**18**	treatment resistant depression/	3,747
**19**	antenatal depression/	846
**20**	perinatal depression/	897
**21**	dysthymia/	9,494
**22**	cyclothymia/	1,109
**23**	(depress* or dysthymi* or cyclothymi* or low mood or mood disorder* or affective disorder*).ti,ab,kw.	739,344
**24**	or/15-23	884,446
**25**	pregnancy/	760,784
**26**	perinatal period/	38,161
**27**	puerperium/	48,716
**28**	puerperal disorder/	5,085
**29**	(pregnan* or ante natal or antenatal or post partum or postpartum or ante partum or antepartum or post natal or postnatal or peri partum or peripartum or peri natal or perinatal or intra partum or intrapartum or puerp* or after birth).ti,ab,kw.	1,056,738
**30**	or/25-29	1,351,677
**31**	24 and 30	40,111
**32**	postnatal depression/	4,757
**33**	31 or 32	41,172
**34**	14 and 33	919

Table S3: Ovid Emcare <1995 to 2021 Week 40>

#	Query	Results from 13 Oct 2021
**1**	behavio* activat*.ti,ab,kw.	1,114
**2**	(behavio* adj3 (reinforce* or re-inforce*)).ti,ab,kw.	1,258
**3**	(behavio* adj2 (contracting or modif*)).ti,ab,kw.	3,813
**4**	reinforc*.ti,kw. or ((positive adj1 reinforc*) or (reinforc* adj3 (environment* or experience*))).ti,ab,kw.	6,543
**5**	(activit* adj2 schedul*).ti,ab,kw.	324
**6**	((pleas* or enjoyable or rewarding) adj3 (activit* or event?)).ti,ab,kw.	813
**7**	((operant or instrumental) adj (conditioning or learning)).ti,ab,kw.	609
**8**	(positive interaction* or avoida* coping or environmental contingenc* or contingency management).ti,ab,kw.	3,137
**9**	functional analysis.ti,ab,kw.	4,008
**10**	behavio*.mp. and (self adj (evaluat* or monitor*)).ti,ab,kw.	2,790
**11**	(behavio* adj (counsel* or intervention* or treatment* or therap* or psychotherap*)).ti,ab,kw.	27,132
**12**	(mood adj3 monitor*).ti,ab,kw.	144
**13**	Behavior Therapy/	15,666
**14**	or/1-13	57,849
**15**	depression/	109,190
**16**	mood disorder/	16,205
**17**	major depression/	24,701
**18**	treatment resistant depression/	798
**19**	antenatal depression/	451
**20**	perinatal depression/	534
**21**	dysthymia/	3,360
**22**	cyclothymia/	377
**23**	(depress* or dysthymi* or cyclothymi* or low mood or mood disorder* or affective disorder*).ti,ab,kw.	196,928
**24**	or/15-23	236,911

**25**	pregnancy/	105,853
**26**	perinatal period/	10,560
**27**	puerperium/	13,321
**28**	puerperal disorder/	265
**29**	(pregnan* or ante natal or antenatal or post partum or postpartum or ante partum or antepartum or post natal or postnatal or peri partum or peripartum or peri natal or perinatal or intra partum or intrapartum or puerp* or after birth).ti,ab,kw.	212,308
**30**	or/25-29	237,711
**31**	24 and 30	13,541
**32**	postnatal depression/	2,480
**33**	31 or 32	14,232
**34**	14 and 33	427

Table S4: APA PsycInfo <1806 to October Week 1 2021>

#	**Query**	**Results from 13 Oct 2021**
**1**	behavioral activation system/	529
**2**	behavio* activat*.tw.	2,515
**3**	(behavio* adj3 (reinforce* or re-inforce*)).tw.	5,721
**4**	exp reinforcement/	53,062
**5**	(behavio* adj2 (contracting or modif*)).tw.	10,147
**6**	(reinforc* or ((positive adj1 reinforc*) or (reinforc* adj3 (environment* or experience*)))).tw.	83,502
**7**	(activit* adj2 schedul*).tw.	645
**8**	Planned Behavior/	2,758
**9**	((pleas* or enjoyable or rewarding) adj3 (activit* or event?)).tw.	1,873
**10**	((operant or instrumental) adj (conditioning or learning)).tw.	6,244
**11**	exp operant conditioning/	36,365
**12**	(positive interaction* or avoida* coping or environmental contingenc* or contingency management).tw.	6,255

**13**	exp contingency management/	3,176
**14**	functional analysis.tw.	3,413
**15**	behavio*.mp. and (self adj (evaluat* or monitor*)).tw.	6,313
**16**	self-management/ and behavior change/	131
**17**	(behavio* adj (counsel* or intervention* or treatment* or therap* or psychotherap*)).tw.	59,429
**18**	(mood adj3 monitor*).tw.	294
**19**	Behavior Therapy/	14,693
**20**	or/1-19	224,468
**21**	major depression/	133,199
**22**	treatment resistant depression/	2,625
**23**	postpartum depression/	5,185
**24**	affective disorders/	14,890
**25**	dysthymic disorder/	1,505
**26**	cyclothymic disorder/	222
**27**	(depress* or dysthymi* or cyclothymi* or low mood or mood disorder* or affective disorder*).tw.	348,935
**28**	or/21-27	353,319
**29**	pregnancy/	24,265
**30**	perinatal period/	3,266
**31**	postnatal period/	5,349
**32**	(pregnan* or ante natal or antenatal or post partum or postpartum or ante partum or antepartum or post natal or postnatal or peri partum or peripartum or peri natal or perinatal or intra partum or intrapartum or puerp* or after birth).tw.	85,318
**33**	or/29-32	86,966
**34**	28 and 33	15,017
**35**	postpartum depression/	5,185
**36**	34 or 35	15,118
**37**	20 and 36	541

Table S5: Cochrane Library

#	Query	Results from 13 Oct 2021
**#1**	behavio* next activat*:ti,ab,kw	978
**#2**	(behavio* Near/3 (reinforce* or re-inforce*)):ti,ab,kw	318
**#3**	(behavio* near/2 (contracting or modif*)):ti,ab,kw	2119
**#4**	reinforc* or ((positive near/1 reinforc*) or (reinforc* near/3 (environment* or experience*))):ti,ab,kw	9878
**#5**	(activit* near/2 schedul*):ti,ab,kw	252
**#6**	((pleas* or enjoyable or rewarding) near/3 (activit* or event?)):ti,ab,kw	403
**#7**	((operant or instrumental) near (conditioning or learning)):ti,ab,kw	382
**#8**	(positive next interaction* or avoida* next coping or environmental next contingenc* or contingency next management):ti,ab,kw	1204
**#9**	"functional analysis":ti,ab,kw	217
**#10**	behavio* and (self near (evaluat* or monitor*)):ti,ab,kw	4174
**#11**	(behavio* near (counsel* or intervention* or treatment* or therap* or psychotherap*)):ti,ab,kw	50818
**#12**	(mood near/3 monitor*):ti,ab,kw	212
**#13**	MeSH descriptor: [Behavior Therapy] this term only	4774
**#14**	#1 or #2 or #3 or #4 or #5 or #6 or #7 or #8 or #9 or #10 or #11 or #12 # or #13	63918
**#15**	MeSH descriptor: [Depression] this term only	13228
**#16**	MeSH descriptor: [Mood Disorders] this term only	855
**#17**	MeSH descriptor: [Depressive Disorder] this term only	8140
**#18**	MeSH descriptor: [Depressive Disorder, Major] this term only	5301
**#19**	MeSH descriptor: [Depressive Disorder, Treatment-Resistant] this term only	481
**#20**	MeSH descriptor: [Dysthymic Disorder] this term only	181

**#21**	MeSH descriptor: [Cyclothymic Disorder] this term only	13
**#22**	(depress* or dysthymi* or cyclothymi* or "low mood " or "mood disorder*" or "affective next disorder*"):ti,ab,kw	93897
**#23**	#15 or #16 or #17 or #18 or #19 or #20 or #21 or #22	94268
**#24**	MeSH descriptor: [Pregnancy] this term only	22835
**#25**	MeSH descriptor: [Peripartum Period] this term only	16
**#26**	MeSH descriptor: [Postpartum Period] this term only	1279
**#27**	MeSH descriptor: [Puerperal Disorders] this term only	312
**#28**	(pregnan* or "ante natal" or antenatal or "post partum" or postpartum or "ante partum" or antepartum or "post natal" or postnatal or "peri partum" or peripartum or "peri natal" or perinatal or "intra partum" or intrapartum or puerp* or "after birth"):ti,ab,kw	80980
**#29**	#24 or #25 or #26 or #27 or #28	80980
**#30**	#23 and 29	7919
**#31**	MeSH descriptor: [Depression, Postpartum] this term only	647
**#32**	#30 or #31	8524
**#33**	#14 and #32	1461
